# Relentless Hypoxia in a Patient With Carcinoid Syndrome

**DOI:** 10.7759/cureus.20497

**Published:** 2021-12-18

**Authors:** Abdulrhman Alghamdi, Afnan A Malibari, Faisal Al-Husayni, Abdullah Jabri, Saad Albugami

**Affiliations:** 1 Internal Medicine, College of Medicine, King Saud Bin Abdulaziz University for Health Sciences, Jeddah, SAU; 2 Internal Medicine, King Saud Bin Abdulaziz University for Health Sciences, Jeddah, SAU; 3 Internal Medicine, King Abdullah International Medical Research Center, King Saud Bin Abdulaziz University for Health Sciences, Jeddah, SAU; 4 Internal Medicine, National Guard Hospital, King Abdulaziz Medical City, Jeddah, SAU; 5 Cardiology, King Abdullah International Medical Research Center, King Saud Bin Abdulaziz University for Health Sciences, Jeddah, SAU; 6 Cardiology, King Faisal Cardiac Center, King Abdulaziz Medical City, Jeddah, SAU

**Keywords:** right to left shunting, carcinoid tumour, hypoxia, valvular heart disease, percutaneous closure, carcinoid heart disease, patent foramen ovale

## Abstract

Patent foramen ovale (PFO) in patients with carcinoid heart disease (CHD) may result in hypoxia due to the presence of large right (R) to left (L) intracardiac shunts leading to hypoxia and worsening clinical condition. Percutaneous closure of the PFO can normalize oxygen saturation, relieve symptoms, and lessens left-sided heart valves involvement.

We describe a case of a 70-year-old female patient with a history of small bowel neuroendocrine tumor on monthly octreotide infusion presented with worsening exertional dyspnea and hypoxia secondary to R to L intracardiac shunt through the PFO. The decision was made to close the PFO percutaneously with Amplatzer (Plymouth, MN: Abbott) PFO occluder device which resulted in immediate normalization of oxygen saturation and relief of her dyspnea.

## Introduction

Carcinoid tumors of the gastrointestinal tract are neuroendocrine tumors that secrete vasoactive substances, mainly serotonin [1]. Vasoactive substances are metabolized by the liver and thus, most patients are asymptomatic [1]. However, patients become symptomatic once hepatic metastasis occurs [1], as these vasoactive substances are not degraded by the liver leading to the development of carcinoid heart disease (CHD) [2]. Similar to the liver, the lungs also metabolize these vasoactive substances which attenuate its effect on the left-sided heart valves [1]. The vasoactive substances promote fibroblast proliferation and plaque deposition leading to endocardial thickening [1-3]. Therefore, resulting in retracted fixed valves and resultant regurgitation, therefore right-sided heart valves are commonly involved in CHD [1-3]. A notable exception is when there is right to left shunt through a patent foramen ovale (PFO) bypassing the lungs, therefore increasing left-sided valve involvement. This may take place in conditions where there is increased right-sided heart pressures (pressure-driven) or if flow is directed towards the PFO (flow-driven) in both conditions systemic hypoxia ensue [1-4].

## Case presentation

A 70-year-old female with a history of small bowel neuroendocrine tumor on monthly octreotide infusion presented with six months history of exertional dyspnea. Her medical history was significant for diabetes mellitus, hypertension, dyslipidemia, and massive pulmonary embolism requiring intensive care unit admission 12 years ago. One week prior to presentation, the exertional dyspnea progressed to the New York Heart Association (NYHA) functional class III-IV. She also complained of chronic diarrhea, loss of appetite, and weight loss. On physical examination, the patient’s blood pressure was 141/71 mmHg, heart rate was 70 beats/minute, respiratory rate was 22 breaths/minute, and temperature was 36.5°C. Oxygen saturation was 85% on room air requiring high-flow oxygen with minimal correction. She was mildly distressed. Chest examination revealed normal air entry with no added sounds. Cardiovascular examination demonstrated normal first and second heart sounds, a pansystolic murmur, and a loud S2. The patient had mild tenderness in the right hypochondrial area on abdominal examination. Arterial blood gas analysis revealed an arterial pH of 7.48, partial pressure of carbon dioxide (PCO_2_) of 31 mmHg, and partial pressure of oxygen (PO_2_) of 83 mmHg.

**Figure 1:**
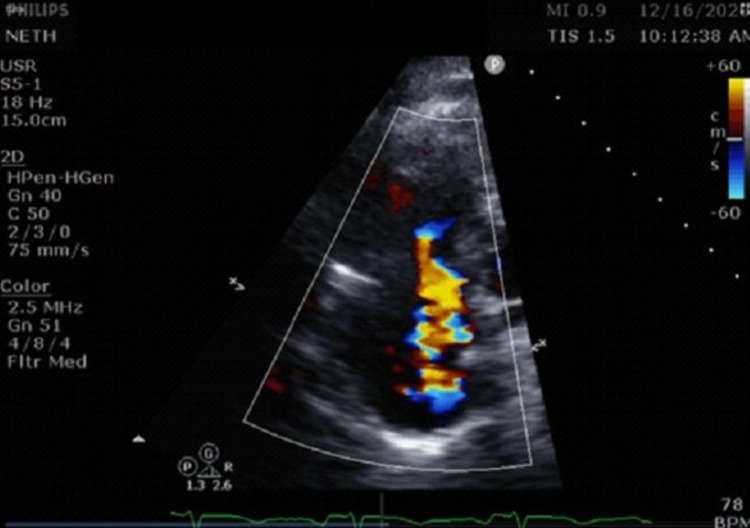
Transthoracic four-chamber view showing severe tricuspid regurgitation.

Computed tomography of the abdomen revealed hypervascular metastases in the liver likely from the previously resected neuroendocrine tumor of the small bowel. Computed tomography pulmonary angiography was negative for pulmonary embolism. Transthoracic echocardiography (TTE) revealed severe tricuspid regurgitation (TR), mild pulmonary hypertension, and a dilated right ventricle with normal systolic function. Degenerative mild to moderate aortic regurgitation (AR) was observed, along with mild to moderate pulmonic valve regurgitation (PR), and normal left ventricular systolic function (Figure 1, Video 1).

**Video 1:** Transthoracic echocardiography revealing severe tricuspid regurgitation.

**Figure 2:**
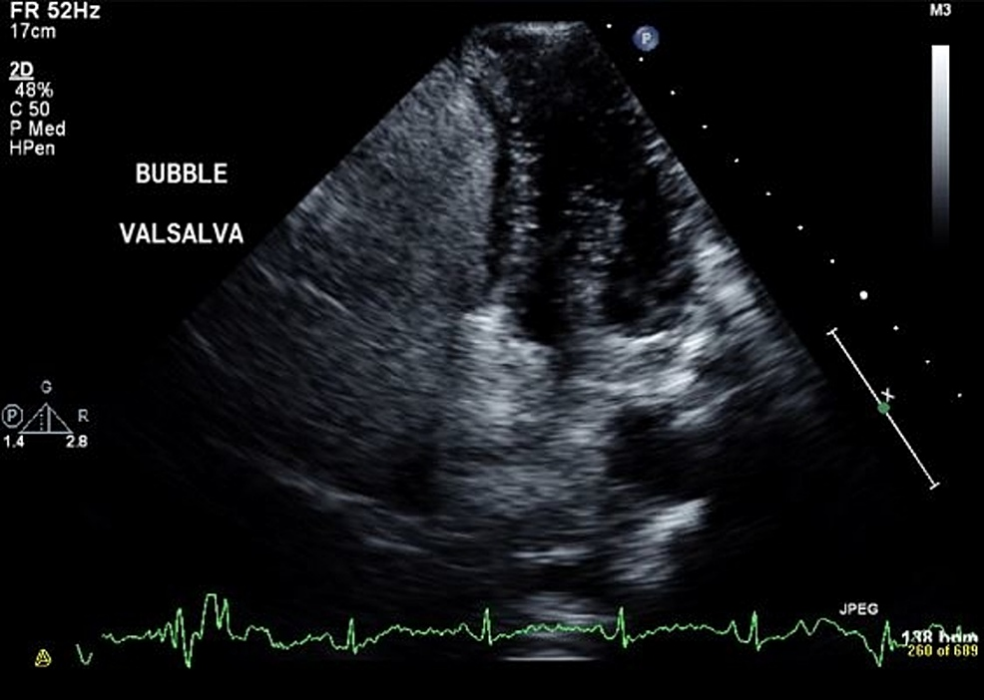
Transthoracic four-chamber view with bubble study demonstrating right to left shunt during Valsalva maneuver.

**Figure 3:**
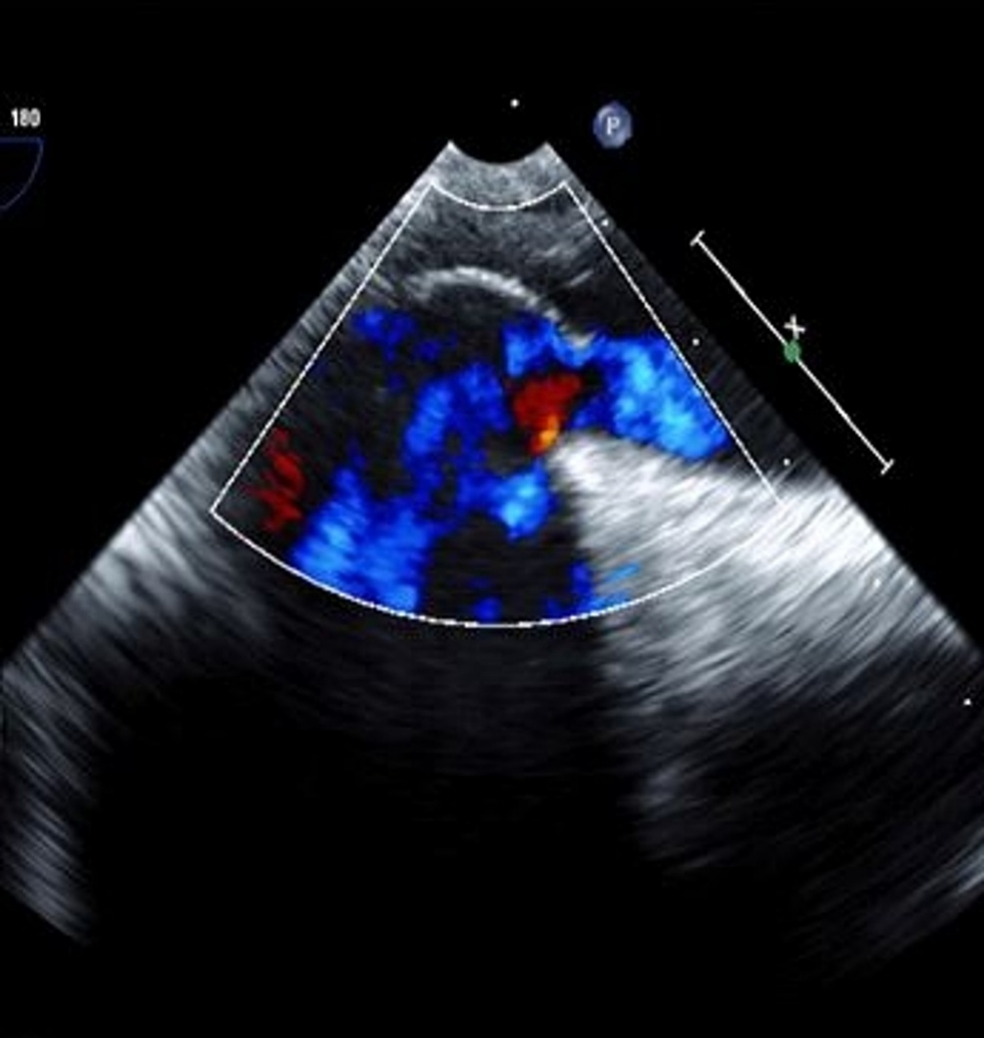
Transesophageal bicaval view (at 90°) showing the tunneled patent foramen ovale with color Doppler demonstrating right to left shunt.

We suspected an intracardiac shunt and performed an echocardiogram with bubble study, which was positive for an interatrial shunt (Figure 2, Video 2). Transesophageal echocardiography (TEE) confirmed the presence of PFO with right to left shunt (Figure 3, Video 3).

**Video 2:** Transthoracic four-chamber view with bubble study demonstrating right to left shunt with microbubbles passing through the patent foramen ovale from the right atrium to the left atrium and ventricle during Valsalva maneuver.

**Video 3:** Transesophageal bicaval view (at 90°) showing the tunneled patent foramen ovale with color Doppler demonstrating right to left shunt.

**Figure 4:**
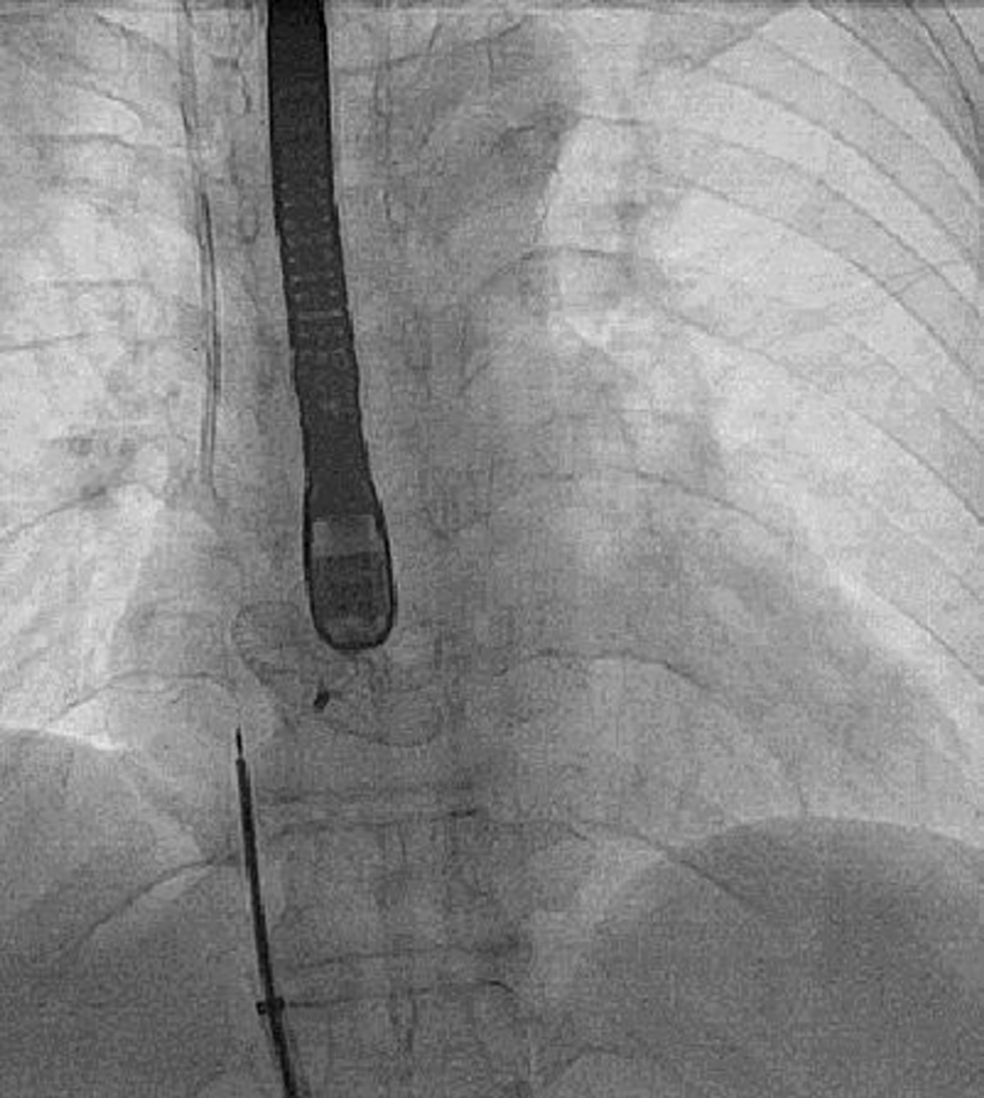
Fluoroscopic view demonstrating closure of the patent foramen ovale.

We then performed left and right heart catheterization which revealed normal coronary arteries. Right heart catheterization revealed normal intracardiac pressures and oxygen step down in the left atrium confirmed a right to left shunt (Table 1). Therefore, we proceeded with closure of the PFO using a 35 mm Amplatzer (Plymouth, MN: Abbott) septal occluder (Figure 4, Video 4).

**Table 1:** Right heart catheterization data. PCWP: pulmonary capillary wedge pressure; LVEDP: left ventricular end-diastolic pressure

Location	Oxygen saturation (%)	Pressure (mmHg)
Superior vena cava	66	-
Inferior vena cava	-	-
Right atrium	65	7
Right ventricle	65	37/0
Pulmonary artery (mean)	65	33/3 (14)
PCWP (mean)	94	(13)
Pulmonary vein	99	-
Left atrium	87	9
Left ventricle	87	10 (LVEDP)

**Video 4:** Coronary angiographic view demonstrating a closure of the patent foramen ovale with a septal occluder.

An intraoperative echocardiogram confirmed complete closure with no residual shunt and oxygen saturation normalized to 95% on room air (Video 5). The patient was observed postoperatively for 24 hours and was later discharged in good health. Three months later, the patient returned with complaints of dyspnea and mild lower limb edema and was treated with escalation of diuresis and referred to surgery for tricuspid and aortic valve surgery.

**Video 5:** An intraoperative echocardiogram confirmed complete closure with no residual shunt.

## Discussion

The incidence of carcinoid syndrome is 50% in patients with preexisting carcinoid tumors [5]. In addition, 20% of patients with carcinoid syndrome develop CHD [6]. Tricuspid and pulmonic valve involvement in right-sided CHD may result in exertional dyspnea, fatigue, and ultimately right-sided heart failure [5-8]. Severe valvular dysfunction in CHD is a major cause of morbidity and mortality [5]. Interestingly, roughly 50% of patients with CHD have a PFO [5]. Thus, a right to left intracardiac shunt should be suspected in patient with CHD and hypoxemia [8,9].

Right to left shunt occurring through the PFO is believed to ensue in two clinical scenarios. The first is due to pressure overload in the right side of the heart caused by many factors (tricuspid stenosis or regurgitation, pulmonary stenosis or regurgitation, pulmonary hypertension, mechanical ventilation, right ventricular ischemia, or infarction). The other scenario takes place in the absence of pulmonary hypertension with normal right-sided heart pressures as in conditions like tricuspid regurgitation directed to the PFO, atrial myxoma, and presence of eustachian valve [4,10].

Many patients can present with platypnea-orthodeoxia syndrome where deoxygenation and dyspnea occur in upright position (orthodeoxia) and is relieved in a supine position (platypnea) this is due to R-L shunting of deoxygenated blood through the PFO, resulting in hypoxemia and dyspnea [8].

We report a case of a carcinoid heart disease with a right to left shunt through a PFO resulting in dyspnea and worsening hypoxia that was successfully treated by percutaneous PFO closure that resulted in normalized oxygen saturation, symptomatic relief, and improved quality of life. To our knowledge, there were only five similar reported cases of flow-driven R-L shunting due to the tricuspid regurgitation jet directing deoxygenated blood towards the PFO, resulting in hypoxemia and dyspnea [8,10-14].

## Conclusions

Systemic hypoxia resistance to normalization with oxygen in patients with PFO suggests significant right to left shunting through a PFO. Ascertaining the mechanism of intracardiac shunts in carcinoid heart disease with PFO is important to accurately diagnose and treat the disease. Percutaneous closure is safe and effective in relieving symptoms, correcting hypoxia, and minimizing left-sided heart valves involvement.
